# Supplementary material for: Effect of discontinuous fair-share emissions allocations immediately based on equity
Source: Nat Commun. 2025 Sep 3;16:8020. doi: 10.1038/s41467-025-62947-9 (PMC12408840; doi:10.1038/s41467-025-62947-9)
Supplement: Supplementary file 1 — Supplementary Information [file 41467_2025_62947_MOESM1_ESM.pdf]

Supplementary Information

For article "**Effect of discontinuous fair-share emissions allocations immediately based on equity**"

Yann Robiou du Pont[1]*, Mark Dekker[1,3], Detlef van Vuuren[1,3], Michiel Schaeffer [1,2,4]

[1] Copernicus Institute of Sustainable Development, Utrecht University, Princetonlaan 8a, 3584 CB Utrecht, Netherlands,

[2] PBL Netherlands Environmental Assessment Agency, Bezuidenhoutseweg 30, 2594 AV Den Haag, Netherlands,

[3] Climate Analytics, Ritterstraße 3, 10969 Berlin, Germany,

[4] Universitas Islam Internasional Indonesia, Jalan Raya Bogor KM. 33.5 Cisalak, Sukmajaya Depok, 16416 West Java Indonesia

* Corresponding author: yann.rdp@climate-energy-college.org

List of Supplementary Items:

Supplementary Discussion

Supplementary Methods

Supplementary Table 1

Supplementary Table 2

Supplementary Figure 1

Supplementary Figure 2

Supplementary References

Supplementary discussion

Funding disclosure
This study was co-funded by Aroha and the Climate Vulnerable Forum (CVF). The CVF requested the development of an analysis reflecting the Paris Agreement's regarding equity and temperature goal. The burden-sharing Approach 1 of the present study was used in a report published by CVF with specific parameterizations using Gross Domestic Product (GDP) and Human Development Index (HDI).

The data presented in the original submission of this manuscript was used in the Traffic Light Reports[1] (https://fairsharenow.org) and is available at https://zenodo.org/record/8003393. The first version includes results for all countries visible as a HyperText Markup Language (HTML) file through different interactive figures. These two files contain emissions allocations data for all countries arranged in five figures, one file with responsibility accounted since 1950, one since 1990. To view the file content, the file needs to be downloaded and can then opened in a web-browser.

The first version includes figures (labelled Fig.1 to Fig. 5 in the file):

- Figure 1 | Greenhouse gas emissions allocation towards 1.5°C. (a) Emissions allocations of the 1.5°C scenario-set are shown in 2030, (b) cumulated budgets over the century, and (c) dynamically.
- Figure 2 | Emissions allocations of various global scenarios from 1.5°C to 4°C, under Approach 1 - GDP (left panel), Approach 1 - HDI (central panel), Approach 2 - GDP (transition) (right panel).
- Figure 3 | Warming assessment of NDCs for various effort-sharing allocations
- Figure 4 | Emissions allocation of the 1.5°C in 2030 under various effort-sharing allocations
- Figure 5 | Differentiated dates for national allocations to equal the global objective of 43% reduction below 2020

Request as explained by the CVF:

"This study was partly funded by Aroha and the Climate Vulnerable Forum (CVF). The CVF secretariat aimed to provide guidance based on over a decade of forum member deliberations on climate policy issues, especially equity considerations, to identify key parameters and concepts that could guide an assessment of NDCs' alignment, or not, with the Paris Agreement temperature goal that might be considered broadly consistent with CVF views. Specifically, the CVF requested that the burden-sharing approach models a combination of a principle of capability and responsibility as per Article 2 of the Paris Agreement.

CVF Parameters for Evaluating NDC Alignment

In order to contribute to discussion and debate on the adequacy of any national climate change mitigation efforts under the Paris Agreement, this paper aims to transparently document key concepts of relevance to the CVF's appreciation of such concerns. Within this context, the following three chief *equity* parameters have, in particular, been proposed to guide this present papers' assessment of all countries NDCs for alignment with the Paris temperature goal:

1. Distribution – the issue of evenly distributing emissions' responsibilities to all countries, whereby everyone has an equal right and responsibility to ensuring a safe climate. This parameter manifests as conferring "common" or shared responsibility to not exceed a given global carbon budget (or, inversely: access rights to this budget) needed to keep within the Paris Agreement temperature goal, implying here country emission allocations are by population scale relative to one another.

2. Interval – the interval or time period over which any countries' per person responsibility for emissions should prevail. The CVF members have generally viewed responsibility to have a historical quality. The text of the UN Framework Convention on climate change (UNFCCC) itself called on developed countries to "lead", noting that "the largest share of historical and current [prior to 1992] global emissions of greenhouse gases has originated in developed countries". The CVF's broader research project is, for now, thereby exploring timeframes to 2100 and commencing in 1990, when the first IPCC report and first UN General Assembly resolution on climate change were adopted, as well as 1950.

3. Capability – the ability of any country to respond to climate change, especially as conditioned by available capacities and resources, which may be measured in a variety of ways, including economic (such as using Gross Domestic Product, GDP) or in human development terms (such using the UNDP Human Development Index).

The mandate provided to experts responsible for the present paper was to resolve the foregoing parameters – and not other – factors in a framework and approach that enabled comparable evaluation of countries' present national climate action pledges (NDCs) with the Paris temperature goal.

Data on developing countries' unconditional and conditional NDCs have been requested in order to contribute and enable discussion, bearing in mind that unconditional NDCs represent what a government is promising to deliver independently, whereas conditional NDCs depend on various forms of international cooperation and support, such as finance, technology, and capacity building."

Interpretation and discussion of CVF's request in light of the available literature:

The three criteria detailed by CVF are comparable to the considerations present in the literature on effort-sharing approaches applied to the Paris Agreement goal, based on capability and responsibility allocations[2–8]. The distribution criterion is reflected in the effort-sharing formula by allocating emissions to countries on a per capita basis, in line with the literature referenced in the manuscript.

The interval criterion is reflected through the accounting for past emissions since 1990 (referred to as “observed” emissions by the CVF) and since 1950 (referred to as “historical” emissions by the CVF). In the absence of consensus under the UNFCCC on a period to account for countries’ responsibilities, the literature referenced in this paper has modelled responsibility for past emissions since 1850, 1950, or starting at current dates. The 1950 and 1990 dates selected here are commonly used in literature.

The capability criterion is reflected in the allocation methods of both approaches 1 and 2 (through GDP), jointly with considerations of responsibility. These considerations are explicitly present in Article 2 of the Paris Agreement and are already modelled in several studies and discussed in the 6th chapter of the Working Group 3 of IPCC AR5. Additional results using HDI as a capability indicator, in addition to GDP, are published in the Traffic Light Report[1], based on this manuscript first submission pre-print[9].

The modelled choices to derive the novel approaches (Approach 1 and Approach 2) were developed by the authors independently of further consideration from CVF and not reviewed by CVF. The manuscript was not written or reviewed by CVF.

Supplementary Methods

Missing data

The unavailability of data necessary to calculate some countries' allocation has a minor impact on the global picture (Supplementary Table 1) and results in the exclusion of some countries in the analysis (Supplementary Table **2**). In this section, countries are referred to by their iso-Alpha 3 codes.

Supplementary Table 1 | Overview of missing data. If we weigh by population, the population data will always be fully complete. Hence, no data in cells filled with (*).

Variable	Percentage of countries missing (Number of countries)	Fraction of population missing	Data source (references in main article)
Population (past)	0% (0)	*	UN population estimates
Population (future)	6.6% (13)	*	SSP2 projections
GDP (future)	8.6% (17)	1.2%	SSP2 projections
Emissions (past)	2.0% (4)	0.065%	PRIMAP[10] + Jones et al. (2023)[11]
NDC	0% (0)	0.%	Climate Resource

Supplementary Table 2 | Countries with missing data. All countries in this table, except for Libya, were excluded from the calculation of emissions allocations as some necessary data is missing. Their allocations are treated as null, which slightly increases the emissions space available to other countries.

Countries with missing variable	Future population	GDP	Emissions
COK (Cook Islands)	X	X	
VAT (Holy See)	X	X	X
MCO (Monaco)	X	X	X
NRU (Nauru)	X	X	
NIU (Niue)	X	X	
SOM (Somalia)			
GMB (The Gambia)			
LIE (Liechtenstein)	X		
PSE (Palestinian Territory, Occupied)		X	X
SMR (San Marino)	X	X	X
MHL (Marshall Islands)	X	X	
LBY (Libya)			
AFG (Afghanistan)		X	
AND (Andorra)	X	X	
DMA (Dominica)	X	X	
PLW (Palau)	X	X	
KNA (Saint Kitts and Nevis)	X	X	
SYR (Syria)		X	
TUV (Tuvalu)	X	X	
VEN (Venezuela)		X	

Additional implications of missing data

- Emissions allocations to groups (e.g., SIDS, LDC, G7) are calculated as the sum of their respective members, even when a few members are missing. The resulting SIDS projections may be biased towards the other island states that are part of SIDS.

- For some countries, the GDP projections become extremely low towards 2100, some of which even have missing values in 2100. This may significantly affect calculations in Approach 2. Some countries obtain an erroneously high share of the total because of these GDP projections. These countries' iso-alpha 3 codes are: "AND", "ATG", "DMA", "GRD", "KIR", "MHL", "FSM", "MCO", "NRU", "PRK", "PLW", "KNA", "SMR", "SYC", "SSD", "TUV", "COK, "VAT", "NIU", SOM", GMB", LIE", PSE".

Monotony of emissions in C-categories

This section discusses the monotony between the warming levels associated with the scenario categories C1, C3, C6 and C8 (1.5°C, 2°C, 3°C and above 4°C rises respectively), and their emissions levels. Even though the positive parts of net-emissions are generally increasing with increasing temperature response, the negative parts of emissions are not always. In Approaches 1 and 2, positive and negative emissions are allocated separately, and may have opposite effects (increasing or decreasing) on the net emissions allocations. These allocations may not monotonously increase along the warming response of the allocated global emissions scenarios. The check is performed on countries as well as country-groups.

For Approach 1, based on GDP, all of the countries and country groups are monotonous in terms of full-century budgets. The monotony check is performed on the 2030 allocations to ensure that the emissions allocations can be used as an ambition metric. For 2030 allocations (accounting for past emissions since 1990), 23 countries have non-monotonous allocations in 2030. For Approach 2, none of the countries have non-monotonous allocations in 2030.

Bunker emissions

Bunker emissions projections were not available for the ensemble of the global emissions scenarios from the AR6 Database[12]. We derived emissions projections for bunker emissions by extrapolating from a range of scenarios of the ELEVATE project (https://www.elevate-climate.org and here).

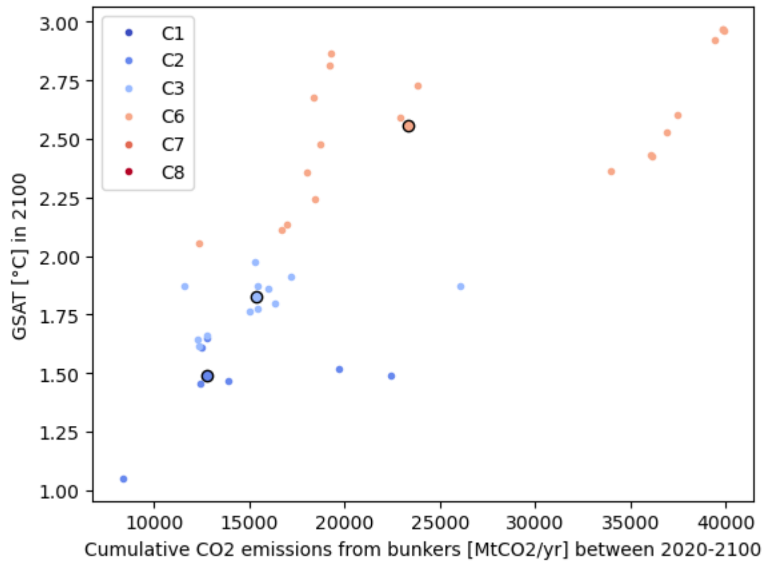

Supplementary Figure 1 | Relationship between 2100 warming of global mean surface air temperature and cumulative CO_2 emissions from bunkers. For each emissions scenario considered, the colour indicates the scenario category (C1-8) reflecting their warming levels, highlighted dots represent the scenario average for each category C2, C3 and C6. Other C-categories, not present in ELEVATE, are obtained through extrapolation (Supplementary Figure 2).

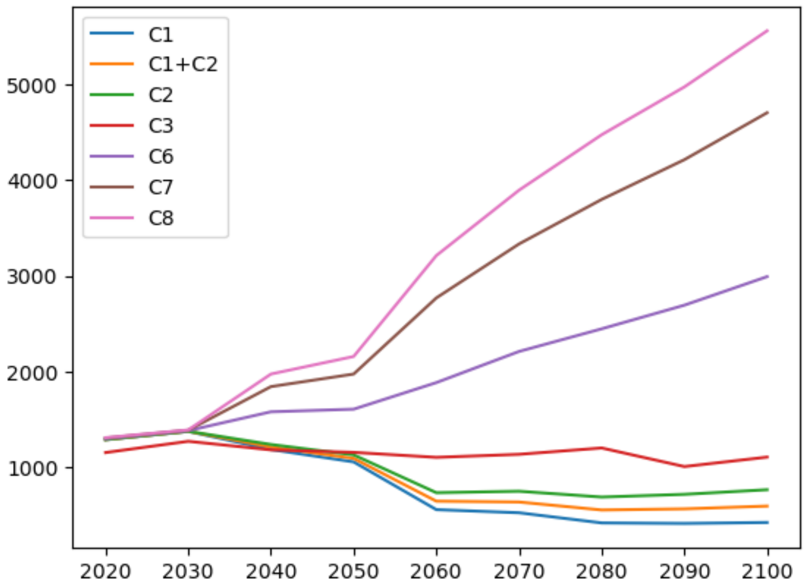

Supplementary Figure 2 | Emissions projections over time (in $MtCO_2e$) for bunker scenarios used in this article based on global emissions scenarios for each category (C1-8) reflecting their warming levels. Bunker emissions extrapolated from the ELEVATE project.

Supplementary References

1. McKinnon, M., Jenkins-Rojas, A., Vásquez, M. J. & Robiou du Pont, Y. *Traffic Light Assessment Report 2023*. https://fairsharenow.org/ (2023).
2. Pan, X., den Elzen, M., Höhne, N., Teng, F. & Wang, L. Exploring fair and ambitious mitigation contributions under the Paris. *Environmental Science and Policy* **74**, 49–56 (2017).
3. Holz, C., Kartha, S. & Athanasiou, T. Fairly sharing 1.5: national fair shares of a 1.5 °C-compliant global mitigation effort. *International Environmental Agreements: Politics, Law and Economics* **18**, 117–134 (2017).
4. Robiou du Pont, Y. *et al.* Equitable mitigation to achieve the Paris Agreement goals. *Nature Climate Change* **7**, 38–43 (2017).
5. Robiou du Pont, Y. & Meinshausen, M. Warming assessment of the bottom-up Paris Agreement emissions pledges. *Nature communications* **9**, 4810 (2018).
6. van den Berg, N. J. *et al.* Implications of various effort-sharing approaches for national carbon budgets and emission pathways. *Climatic Change* **162**, 1805–1822 (2020).
7. Fyson, C. L., Baur, S., Gidden, M. & Schleussner, C. F. Fair-share carbon dioxide removal increases major emitter responsibility. *Nature Climate Change* **10**, 836–841 (2020).
8. Dekker, M. M. *et al.* Navigating the black box of fair national emissions targets. *Nat. Clim. Chang.* **15**, 752–759 (2025).
9. Robiou du Pont, Y., Dekker, M., van Vuuren, D. & Schaeffer, M. Effect of discontinuous fair-share emissions allocations immediately based on equity. *Nature Communications (upcoming, accepted July 2025)* (2025) doi:10.5281/ZENODO.8003393.
10. Gütschow, J. & Pflüger, M. The PRIMAP-hist national historical emissions time series v2.5 (1750-2022). *Zenodo* (2023).
11. Jones, M. W. *et al.* National contributions to climate change due to historical emissions of carbon dioxide, methane, and nitrous oxide since 1850. *Sci Data* **10**, 155 (2023).
12. Byers, E. *et al.* AR6 Scenarios Database. (2022) doi:10.5281/zenodo.5886912.
