# Supplementary material for: Effect of discontinuous fair-share emissions allocations immediately based on equity
Source: Nat Commun. 2025 Sep 3;16:8020. doi: 10.1038/s41467-025-62947-9 (PMC12408840; doi:10.1038/s41467-025-62947-9)
Supplement: Supplementary file 2 — Description of Addtional Supplementary File [file 41467_2025_62947_MOESM2_ESM.pdf]

Description of Additional Supplementary File

Supplementary Data 1 - Effect of discontinuous fair-share emissions allocations immediately based on equity.
