# Supplementary figures and images for: Effect of discontinuous fair-share emissions allocations immediately based on equity
Source: Nat Commun. 2025 Sep 3;16:8020. doi: 10.1038/s41467-025-62947-9 (PMC12408840; doi:10.1038/s41467-025-62947-9)

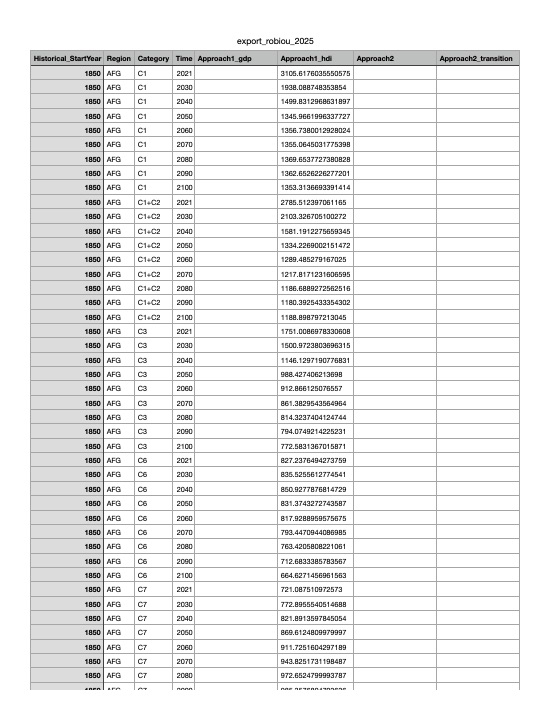

Supplement: Supplementary file 3 — Supplementary Data 1 [file 41467_2025_62947_MOESM3_ESM.zip › preview.jpg]

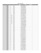

Supplement: Supplementary file 3 — Supplementary Data 1 [file 41467_2025_62947_MOESM3_ESM.zip › preview-micro.jpg]

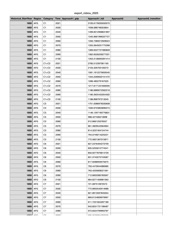

Supplement: Supplementary file 3 — Supplementary Data 1 [file 41467_2025_62947_MOESM3_ESM.zip › preview-web.jpg]
